# Self-Assembling Peptide SCIBIOIII Hydrogel for Three-Dimensional Cell Culture That Promotes Wound Healing in Diabetic Mice

**DOI:** 10.3390/gels9040265

**Published:** 2023-03-23

**Authors:** Lu He, Shijian Lan, Qingfeng Cheng, Zhongli Luo, Xuemei Lin

**Affiliations:** 1 College of Basic Medical Science, Chongqing Medical University, Chongqing 400016, China; 2 The Chongqing Key Laboratory of Translational Medicine in Major Metabolic Diseases, The First Affiliated Hospital of Chongqing Medical University, Chongqing 400016, China; 3 Molecular Medicine and Cancer Research Center, College of Basic Medical Science, Chongqing Medical University, Chongqing 400016, China; 4 Department of Endocrinology, The First Affiliated Hospital of Chongqing Medical University, Chongqing 400016, China

**Keywords:** self-assembling peptide, SCIBIOIII, cellular 3D culture, diabetic wound

## Abstract

An important clinical challenge is improving the healing rate of diabetic chronic wounds, and developing new approaches that can promote chronic wound healing is essential. A new biomaterial that has demonstrated great potential for tissue regeneration and repair is self-assembling peptides (SAPs); however, they have been less studied for the treatment of diabetic wounds. Here, we explored the role of an SAP, SCIBIOIII, with a special nanofibrous structure mimicking the natural extracellular matrix for chronic diabetic wound repair. The results showed that the SCIBIOIII hydrogel in vitro has good biocompatibility and can create a three-dimensional (3D) culture microenvironment for the continuous growth of skin cells in a spherical state. The SCIBIOIII hydrogel in diabetic mice (in vivo) significantly improved wound closure, collagen deposition, and tissue remodeling and enhanced chronic wound angiogenesis. Thus, the SCIBIOIII hydrogel is a promising advanced biomaterial for 3D cell culture and diabetic wound tissue repair.

## 1. Introduction

Diabetes mellitus (DM) is a serious systemic metabolic disease characterized by high blood glucose levels for a long time, resulting in a series of complications [1]. The high diabetes mellitus prevalence is a threat to human health and life worldwide [2]. Changes in the physiological mechanisms of patients can occur owing to long-term persistent hyperglycemia, which, in turn, results in the delayed or even nonhealing of damaged skin and surrounding soft tissues, causing chronic ulcer formation, particularly in the foot and even leading to limb amputation [3]. The classical clinical treatment approaches comprise surgical debridement, dressing, wound decompression, and negative pressure wound therapy [4,5]. However, “passive dressings”, such as gauze, are clinically inefficient for chronic complex wound treatments because they cannot adjust to the microenvironmental modifications of the wound surface or continually slow-release medicines to enhance wound healing [6]. Consequently, new wound dressings to accelerate diabetic wound tissue regeneration are critically needed.

Hydrogels are a very promising biomaterial owing to their several beneficial functions, such as excellent moisturization, permeability, biocompatibility, degradability, and drug transportation [7,8]. Self-assembling peptides (SAPs) are functional polymer nanomaterials synthesized from natural amino acids [9]. Additionally, synthetic peptides, including nucleopeptides [10], are able to self-assemble, leading to important biological properties [11,12]. Nucleopeptides are currently being investigated for the innovative opportunities offered for tissue engineering applications [13]. Under certain conditions, SAPs can spontaneously form crosslinked nanofiber-rich hydrogels. For example, under salt ion conditions, RADA16 and KLD12 can rapidly form permeable hydrogels containing nanofiber structures with more than 99% water content [14,15]; SAPs have found extensive use across many industries since the discovery of SAPs by Zhang [16] and others. SAP hydrogels can mimic the function of the extracellular matrix (ECM) in the area of regenerative repair, and a beneficial cellular microenvironment for tissue regeneration is provided by this [17]. Although the function of SAPs in promoting wound repair has been described, the effect of SAPs alone in the treatment of diabetic wounds has been studied less, and its effectiveness and mechanism of action have not been fully demonstrated.

In this study, a novel self-assembled peptide, SCIBIOIII, is reported. First, we observed its gel-forming ability. In order to provide a 3D microenvironment for cell growth, we cultured mouse skin fibroblasts, in vitro, in a hydrogel formed by SCIBIOIII and analyzed the growth activity of the fibroblasts in the SAP hydrogel. Subsequently, a diabetic mouse wound model was prepared, and the SCIBIOIII hydrogel was applied to the damaged skin of the mice, and the wound healing rate, wound histomorphological characteristics, collagen formation, and angiogenesis after SCIBIOIII treatment were assessed.

## 2. Results and Discussion

### 2.1. Physicochemical Characterization of SCIBIOIII

#### 2.1.1. SCIBIOIII Can Assemble to Form Nanofibrous Structures

**Figure 1:**
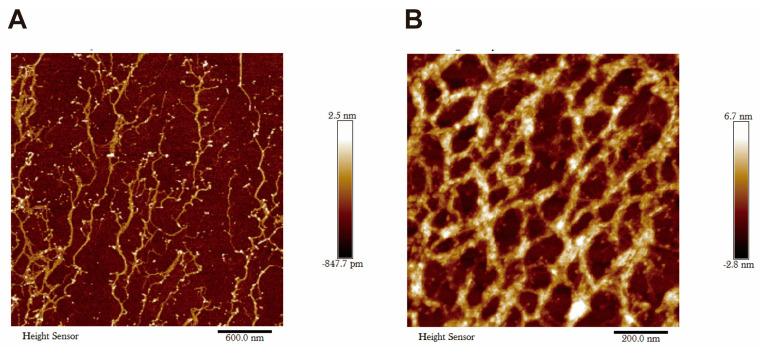
Microscopic morphology analysis of SCIBIOIII.AFM morphology of SCIBIOIII at 24 h. (**A**) AFM morphology of SCIBIOIII at 600 nm. (**B**) AFM morphology of SCIBIOIII at 200 nm.

The observation of the SCIBIOIII microstructure under AFM shows that after 24 h of assembly, the peptide forms many ordered, dense nanofiber structures that intertwine to form a nanofiber network scaffold (Figure 1).

#### 2.1.2. SCIBIOIII Forms a Fibril-Like Membrane Sheet Structure upon the Addition of PBS

**Figure 2:**
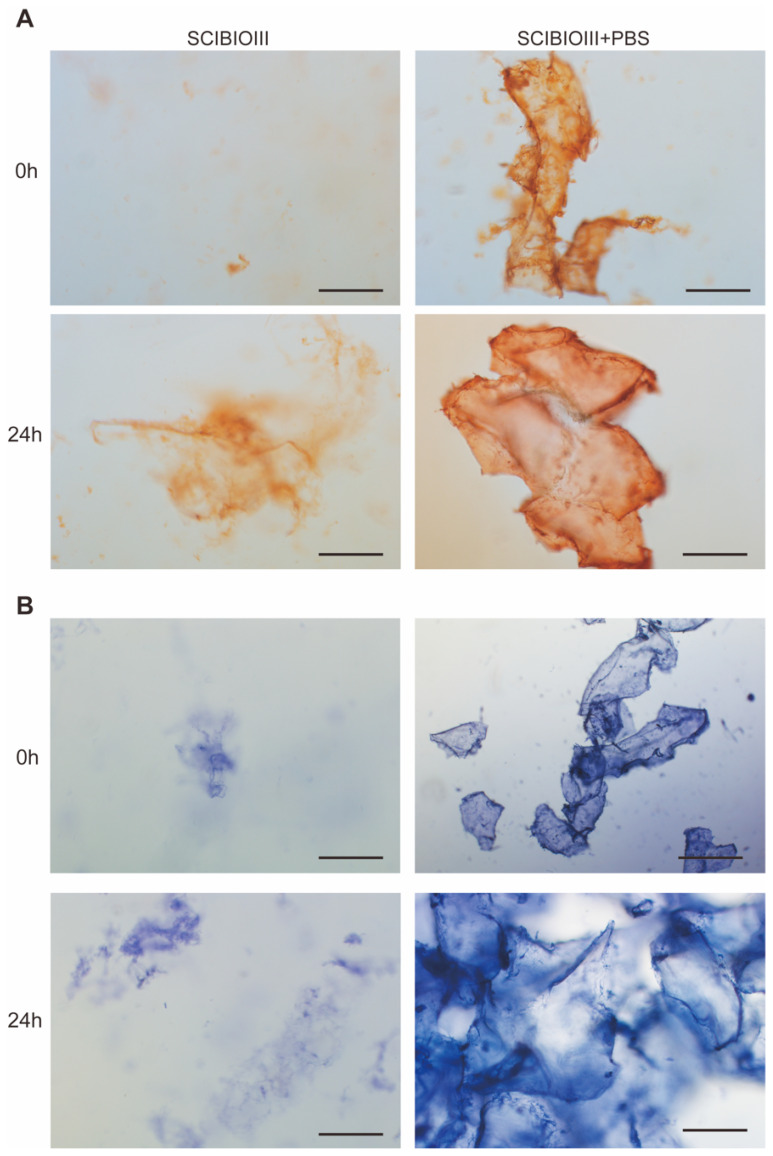
Congo red/aniline blue staining analysis. (**A**) Congo red staining results of SCIBIOIII at 0 h and 24 h; (**B**) aniline blue staining results of SCIBIOIII at 0 h and 24 h. Scale bar: 200 µm.

The results of Congo red/aniline blue staining demonstrated that the SCIBIOIII structure without the addition of PBS was quicksand−like and diffusely sparse at 24 h, while after adding PBS to trigger self-assembly for 24 h, it aggregated into a dense and stable fragment-like structure with clear boundaries, similar to a fibrous mesh structure, which is conducive to the construction of a 3D environment for cell growth (Figure 2).

### 2.2. Cell 3D Culture

#### 2.2.1. SCIBIOIII Successfully Established a 3D Cell Culture System

**Figure 3:**
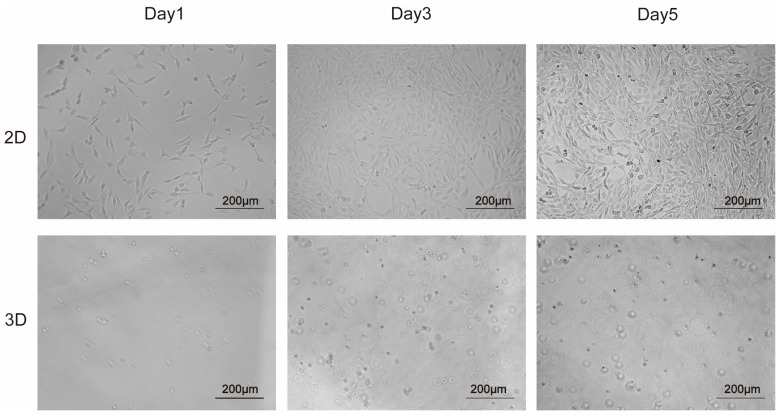
Microscopic morphology of NIH3t3 at days 1, 3, and 5 in 2D and 3D cultures. The 2D group did not have peptide added, and the 3D group had 50 µL of SCIBIOIII peptide solution added in each well.

Under an ordinary light microscope, the mouse skin fibroblasts can be observed under conventional culture conditions in a long shuttle shape and grow in a single layer against the wall. In contrast, mouse skin fibroblasts grew in a round spherical shape and multi−layered space in the hydrogel formed by SCIBIOIII. The results showed that the SCIBIOIII hydrogel could create a 3D microenvironment for NIH3T3 cell growth (Figure 3).

#### 2.2.2. Cell Cytotoxicity in the SCIBIOIII Hydrogels

**Figure 4:**
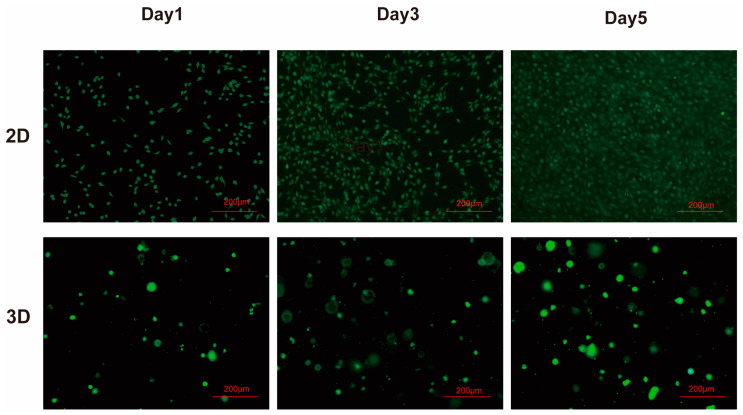
Cytotoxicity analysis of NIH3t3 in SCIBIOIII hydrogels. Calcein-AM staining analysis of NIH3t3 cells cultured in 2D and 3D on days 1, 3, and 5. Live cells are green.

Further, we observed that mast live cells were growing in a circular shape in the 3D environment via live/dead staining assays, and the number of live cells in the 3D environment gradually increased with the increase in culture time. These experimental results indicate that NIH3t3 cells can grow in the 3D environment constructed by SCIBIOIII, which has good biocompatibility and no cytotoxicity (Figure 4).

### 2.3. Animal Experiments

#### 2.3.1. SCIBIOIII Accelerates Wound Closing in Diabetic Mice

**Figure 5:**
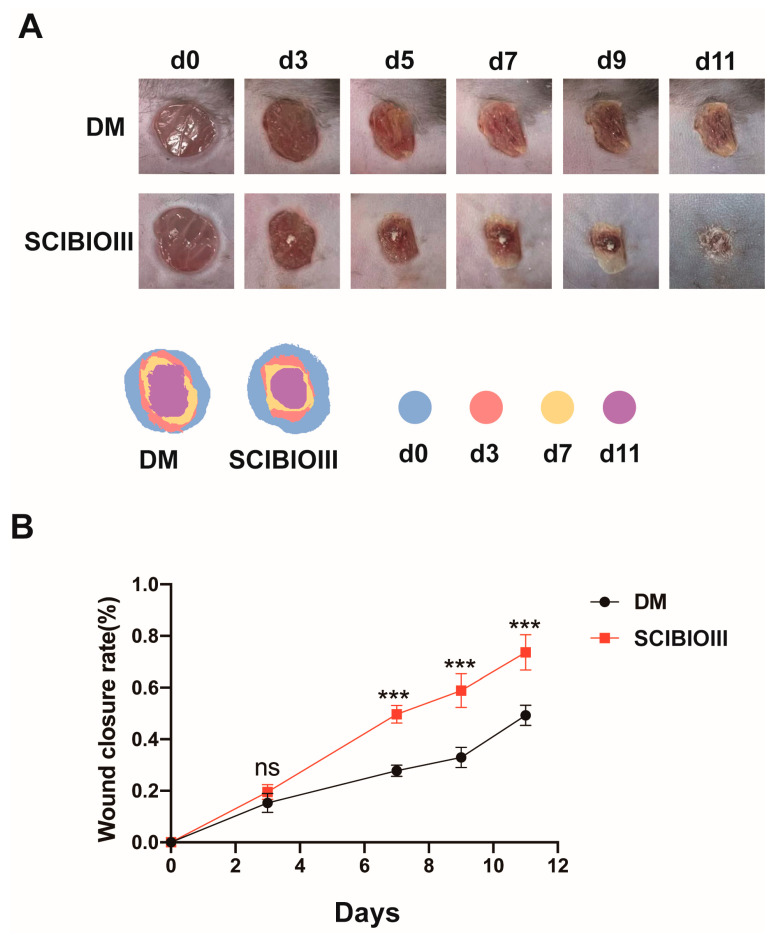
SCIBIOIII hydrogel accelerates wound closure in diabetic mice. (**A**) Photographs of skin wounds (representative images) and signs of wound closure within 11 days were recorded at the specified times (*n* = 5). (**B**) Wound recovery rates from day 0 to 11 were calculated based on images of the wounds (*n* = 10 wounds). ns *p* > 0.05, *** *p* < 0.001.

The impact of the SCIBIOIII hydrogel on chronic wounds was first assessed in the diabetic mouse trauma model. By photographing the back wounds of both groups of mice at the indicated times, it was found that from the 5th day after treatment, the SCIBIOIII group had significantly higher wound healing than the diabetic group (Figure 5A). Further, our quantitative analysis of the area size of the wounds in both groups of mice on days 7, 9, and 11 of the healing process showed that the SCIBIOIII hydrogel-treated group significantly increased the wound closure rate in diabetic animals compared to the model group (Figure 5B).

#### 2.3.2. SCIBIOIII Promotes Granulation Tissue Formation on Wounds

**Figure 6:**
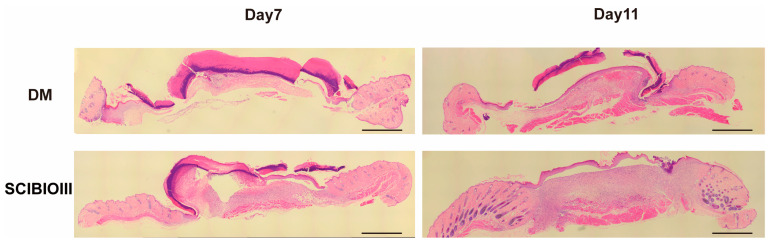
SCIBIOIII hydrogel advanced the formation of granulation tissue in diabetic mice with skin wounds. Representative pictures on days 7 and 11 (*n* = 5). Scale bar: 1.5 mm.

Subsequently, we performed morphological analysis of wound tissue in diabetic mice. The granulation tissue formation is an important index to evaluate the degree of wound healing. The HE staining results showed that the skin wound in the DM group was significantly wider than that in the SCIBIOIII group on the 7th day after treatment, and no obvious new granulation tissue was observed, while obvious granulation tissue and new epidermis were observed in the hydrogel injection group. Notably, on postoperation day 11, the degree of wound healing was significantly higher in the DSCIBIOIII group than that in the model group, and the skin epidermis and dermis were more intact and orderly (Figure 6).

#### 2.3.3. SCIBIOIII Promotes Collagen Synthesis in Diabetic Mice Skin Trauma Tissue

**Figure 7:**
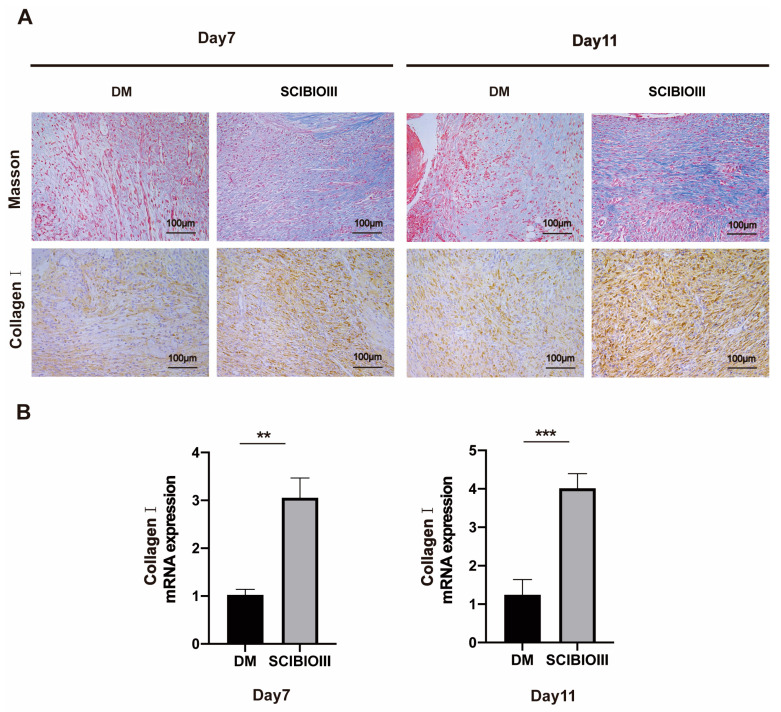
SCIBIOIII hydrogel promotes collagen formation in diabetic mice. (**A**) Masson’s trichrome staining and immunohistochemical analysis of collagen on days 7 and 11 in the skin tissue damaged by diabetic mice injected with or without SCIBIOIII (representative images) (**B**) Detection of mRNA expression of collagen I in skin wounds of diabetic mice treated or untreated with SCIBIOIII on days 7 and 11 by real-time PCR (*n* = 5). ** *p* < 0.01, *** *p* < 0.001.

Next, collagen deposition changes in the skin samples treated with and without the SCIBIOIII hydrogel were examined. MASSON staining demonstrated a significant increase in collagen deposition in diabetic skin wounds on days 7 and 11 after SCIBIOIII hydrogel injection compared to the DM group without SCIBIOIII treatment (Figure 7A). Moreover, the IHC staining results also showed a significant increase in collagen I expression on days 7 and 11, following surgery, in the wounds of the peptide group (Figure 7A). Furthermore, the real−time fluorescence quantitative PCR results revealed that the ECM protein collagen I mRNA expression volumes in the wounds of the SCIBIOIII group were significantly higher than in the DM model group (Figure 7B). These results demonstrate that the formation of collagen in diabetic skin tissue is enhanced when using SCIBIOIII hydrogel.

#### 2.3.4. SCIBIOIII Promotes Wound Angiogenesis

**Figure 8:**
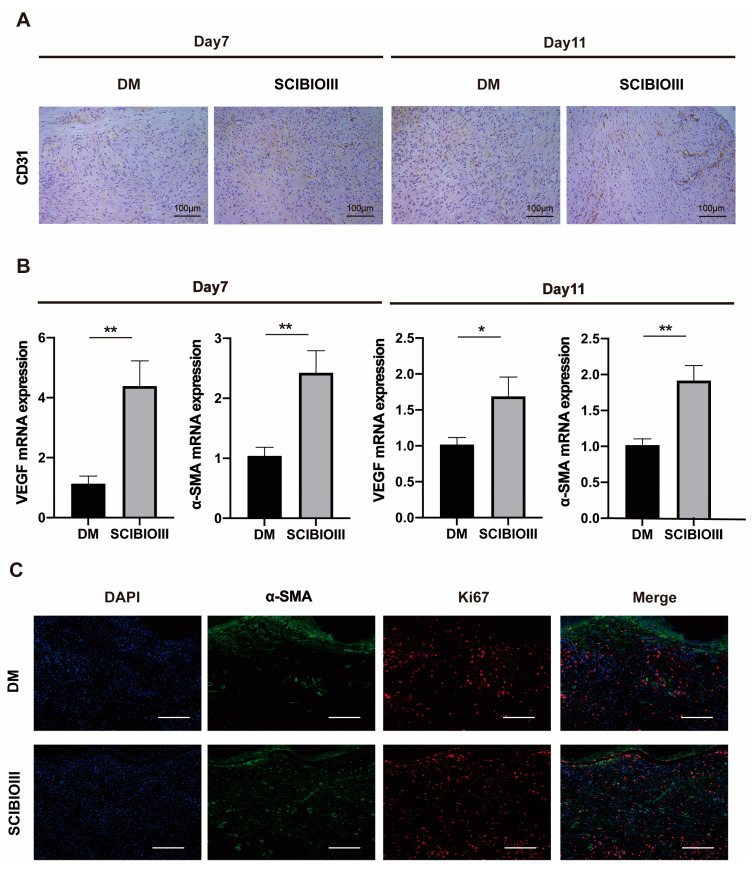
(**A**) IHC results of CD31 from days 7 and 11 from the skin tissue of diabetic mice treated or untreated with SCIBIOIII (representative images). (**B**) qPCR results, the mRNA levels of VEGF and α−SMA on days 7 and 11 from the skin tissue of diabetic mice treated or untreated with SCIBIOIII by qPCR (*n* = 5). (**C**) Immunofluorescence staining results of skin tissue sections on day 11. Scale bar: 100 µm. * *p* < 0.05, ** *p* < 0.01.

Finally, the effect of the SCIBIOIII hydrogel on diabetic wound angiogenesis was also analyzed. First, IHC was used to stain the wound tissue of each group for the vascular endothelial cell marker CD31. The results revealed that the wound tissue of the SCIBIOIII group displayed an obvious increase in CD31 expression compared to the model group on days 7 and 11 following surgery, and a greater number of new capillaries was observed (Figure 8A). Furthermore, significant differences between the two groups were observed using the mRNA expression levels of classic markers: the vascular endothelial growth factor (VEGF) and α-SMA, which is used to evaluate neovascularization, and the treatment group was significantly higher than the model group (Figure 8B). As expected, the expression levels of α−SMA and Ki67 (cell proliferation marker) in the hydrogel injection group using the immunofluorescence staining results of the skin tissue sections on day 11 showed significantly higher results than those in the untreated group (Figure 8C). This provides further evidence that hydrogels can enhance angiogenesis. These results suggest that the SCIBIOIII hydrogel can promote de novo capillary angiogenesis in chronic wound healing.

### 2.4. Discussion

Wound healing is an intricate and dynamically changing process. The wound healing process primarily consists of four overlapping steps: hemostasis, inflammation, proliferation, and, finally, wound remodeling. During these stages, multiple cells and growth factors interact together to participate in wound repair [1]. Patients with diabetes are chronically hyperglycemic, resulting in altered physiological mechanisms resulting in chronic nonhealing wounds owing to insufficient angiogenesis, impaired collagen deposition, and little re−epithelialization during the restoration period of wound healing. An important clinical challenge is the effective promotion of diabetic wound healing. Recently, hydrogel-based dressings have shown powerful effects in accelerating trauma-repairing in animal experiments and clinical trials [15,18,19]. Promising results have been shown by SAP hydrogels when promoting the regenerative repair of nerve injuries [20], cartilage regeneration [21], and skin wound healing [22], owing to the unique advantage of mimicking natural ECM. However, the ability of SAP hydrogel therapy alone to treat diabetic wounds still needs to be explored. In this report, we developed a mouse model of diabetic wounds with streptozotocin to determine whether SCIBIOIII accelerates diabetic wound healing. Our results demonstrated that SCIBIOIII hydrogel treatment increased overall wound repair efficiency, including quicker wound closure rate, greater angiogenesis, increased production of the ECM, and the re-epithelialization of diabetic wounds in mice. Therefore, these results demonstrate that the SCIBIOIII hydrogel is a potential bioactive material for diabetic wound healing.

SAP hydrogels can imitate the natural microenvironment of cell growth in vivo and have been designed as scaffolding materials for 3D cell culture and tissue engineering [23]. The microstructure of SCIBIOIII was observed as thicker nanofibers under atomic force microscopy, and Congo red and aniline blue staining revealed the formation of a visible membrane sheet-like structure upon the self-assembly of SCIBIOIII. The further successful 3D culture of NIH3T3 cells in the hydrogel formed by SCIBIOIII demonstrated the good biocompatibility of SCIBIOIII, with a good cell growth state. In conclusion, our experiments indicated that the wound-healing-promoting effect of SCIBIOIII may be attributed to its ability to form a unique 3D nanofiber network structure on the wound surface. An appropriate microenvironment for the growth of cells in wound repair can be provided because the scaffold formed by these interlaced nanofibers has the function of the ECM.

The ECM is a noncellular 3D macromolecular network comprising collagen, proteoglycan/glycosaminoglycan, elastin, fibronectin, laminin, and several other glycoproteins [24]. ECM remodeling is essential for wound healing; however, ECM synthesis and degradation balance are largely disrupted in diabetic wounds [25]. Diabetic wounds typically exhibit high reactive oxygen species (ROS) levels and enhanced ECM degradation owing to elevated levels of matrix metalloproteinases [26]. A crucial component of the ECM is collagen, which provides strength, establishes the framework of the normal tissues, and participates in the repair of defects caused by injury, thereby facilitating tissue structure and function restoration [27,28]. The production and formation of collagen I are critical in the maturation process of wound repair [24,29]. Therefore, our study tested the protein and mRNA expression levels of collagen I in traumatized tissues. Undoubtedly, higher collagen I levels were found in the tissue samples of diabetic mice injected with the SCIBIOIII hydrogel than in the model group, indicating that the SCIBIOIII hydrogel promotes the synthesis of ECMs in the wounds of diabetic mice.

A major issue in the recovery of chronic diabetic wounds is inadequate blood supply, manifesting primarily as insufficient angiogenesis and decreased granulation tissue formation [30]. Neovascularization significantly improves the trauma microenvironment and facilitates trauma closure. New tissue will not grow or repair the wound unless an adequate blood supply exists for the direct exchange of oxygen, nutrients, and waste products [31]. SAP can be engineered with angiogenic bioactivities as a tailored material to promote neovascularization [32,33,34]. CD31 is a typical indicator of neovascularization. VEGF is crucial in controlling angiogenesis [35]. Multiple experiments in this research confirmed that the expression of CD31, VEGF, and α-SMA in the wound tissue of diabetic mice using the SCIBIOIII hydrogel was higher, and there were more new blood vessels than that in the other group. It was shown that the SCIBIOIII hydrogel could accelerate wound healing by promoting angiogenesis.

In summary, our results confirmed that under ion-triggered conditions, the SCIBIOIII used in this study can form a nanofibrous network and has good biocompatibility for 3D cell culture. Moreover, it can spontaneously form a hydrogel to create a microenvironment with high water content at the wound site that mimics the ECM and accelerates diabetic chronic wound healing by promoting ECM remodeling and angiogenesis. However, this study failed to explore the specific molecular mechanism of its promotion of diabetic wound healing; secondly, wound healing in animal experiments was only observed up to day 11, while complete wound healing was not observed. Additionally, inflammation is also a challenge affecting diabetic wound healing; therefore, in future studies, whether SCIBIOIII can control diabetic wound inflammation to further accelerate chronic wound healing must be investigated.

## 3. Conclusions

In our study, we used a novel self-assembling peptide to investigate its role in chronic diabetic wound healing. Our results demonstrate that this peptide has good gel-forming properties and is able to rapidly change from liquid to gel on the wound surface. Moreover, this peptide possesses good biocompatibility and can be used in cellular and animal experiments. Importantly, in animal experiments, we observed a striking accelerated chronic wound healing effect. Thus, this peptide is a very valuable biomaterial with promising applications in the biomedical field. In conclusion, our study provides a new therapeutic strategy for clinical practitioners.

## 4. Materials and Methods

### 4.1. Dissolve SCIBIOIII

A total of 1 mL of double−distilled water was added to 10 mg SCIBIOIII lyophilized powder with 1 mL disposable syringe in the ultra-clean table, mixed thoroughly, and stored at 4 °C.

### 4.2. Atomic Force Microscopy (AFM)

The surface microscopic topography and structure information of SCIBIOIII was obtained by AFM. Briefly, we added 10 µL PBS into 10 µL peptide solution (10 mg/mL), assembling for 24 h at 37 °C, acquiring images under AFM.

### 4.3. Congo Red/Aniline Blue Staining

A total of 10 mg/mL SCIBIOIII solution was mixed in the ratio of 1:1 with PBS; moreover, Congo red/aniline blue dye was added after assembly at 0 h and 24 h. A microscope was used to observe the morphology of SCIBIOIII.

### 4.4. Construction of a Three-Dimensional Culture System for NIH3t3 Cells

High glucose DMEM medium with 10% FBS was used to cultivate NIH3t3 cells (Sangon Biotech, Shanghai, China) in a 37 °C incubator with 5% CO_2_. The 3rd–5th generation cells were harvested in good growing conditions. The cell concentration was adjusted to 2 × 104 cells, and the cells were resuspended with 1 mL of 10% sucrose solution. The peptide solution was quickly blended with the sucrose/cell mixture at a volume ratio of 1:3 on 96-well plates. A total of 100 µL of high glucose DMEM complete medium was added to each well, cultured at 37 °C in an incubator with 5% CO_2_. Photographs were recorded under the microscope on days 1, 3, and 5.

### 4.5. Cell Live/Dead Staining

The cells were assayed for growth activity in the hydrogel on days 1, 3, and 5. The complete medium was discarded to add PBS to wash the cells 2–3 times. In the dark, the prepared Calcein−AM staining solution (Beyotime, Shanghai, China) 100 µL was added to each well and incubated at 37 °C for 25 min. The pictures of stained cells were recorded using a fluorescence microscope.

### 4.6. Diabetic Wound Model

Wild-type (WT) C57BL/6 male mice (Chongqing ENSIWEIER Biotechnology Co., Ltd, China), aged 7–8 weeks, were habitually housed in an SPF−grade animal laboratory at a temperature of (21 ± 1) °C, at a relative humidity of 55 ± 5%, with 12 h of translating light and dark, and free drinking water. After dissolving 50 mg/kg Streptozotocin (Solarbio, Beijing, China) in citrate buffer (0.1 mol/L, pH 4.5), it was injected into mice’s abdomen for five consecutive days. Blood glucose levels were tested by glucometer (Sinocare, Hunan, China) after one week. Mice with random blood glucose concentration >16.7 mmol/L and weight loss were diagnosed with diabetes mellitus. Mice with diabetes were anesthetized using Isoflurane after two weeks. After shaving and disinfection, using a skin biopsy perforator (Acuderm, Florida, USA) made two 6 mm diameter total excision wounds on the back of each one. Two groups of the model mice were randomly selected (*n* = 5), and each group was treated using 20 µL of PBS or SCIBIOIII (10 mg/mL) for three consecutive days.

### 4.7. Wound Healing Assessment

Digital photographs of mouse wounds were collected on days 0, 3, 5, 7, 9, and 11 following surgery to evaluate the wound healing process. ImageJ software was used to determine the size of the wound area. The wound closure rate was calculated by subtracting the wound area at the specified time from the initial wound area. On days 7 and 11 after surgery, the samples were divided into two parts: one part was placed in a preservation tube filled with 4% paraformaldehyde for histological analysis, and the other part was put into liquid nitrogen quick-frozen for qPCR analysis and placed in an ultra−low temperature refrigerator.

### 4.8. Morphological Analysis

Hematoxylin and eosin (HE) staining was used to evaluate the degree of wound healing; Masson staining was used to evaluate the collagen formation of skin wounds; while immunohistochemical and immunofluorescence stainings were used to analyze new blood vessels, collagen deposition, and wound tissue cell proliferation. The intact skin wound tissue was fixed in 4% paraformaldehyde for HE staining and Masson staining, was dehydrated, embedded in paraffin, sectioned, and stained using HE staining solution (Scientist biotechnology, Sichuan, China) or Masson staining solution according to the kit instructions. For immunohistochemical staining (IHC) staining, skin tissue sections were prepared as before, antigen retrieval was performed on the sections, and target antibodies CD31 and collagen I or fluorescently labeled antibodies Ki67 and a−SAM were added and incubated overnight. Following this secondary antibodies were added to incubate for color development, and then hematoxylin was used to restain the nuclei. Following staining and slide mounting, images were acquired under a light microscope or a fluorescence microscope.

### 4.9. qPCR Analysis

Using a purchased commercial kit (AccurateBiology, Hunan, China), total RNA was extracted from the sample and reverse transcribed into cDNA. SYBR Green was used as a fluorescent marker. Target gene primer sequences were provided in Table S1. After the evaluation data fulfilled the requirements, the relative expression level of the target gene was calculated using the delta-delta CT method.

### 4.10. Statistical Analysis

The data were all given as mean SEM. Two independent-sample *t*-tests were used to examine the differences between the groups. Data were analyzed using the GraphPad Prism program (version 9.0). All trials in this paper were repeated three or more times. *p* < 0.05 was regarded as statistically significant.

## Data Availability

The data presented in this study are available in the article.

## Supplementary Materials

The following supporting information can be downloaded at: https://www.mdpi.com/article/10.3390/gels9040265/s1, Table S1: Primer sequence.
