# Supplementary material for: Self-Assembling Peptide SCIBIOIII Hydrogel for Three-Dimensional Cell Culture That Promotes Wound Healing in Diabetic Mice
Source: Gels. 2023 Mar 23;9(4):265. doi: 10.3390/gels9040265 (PMC10137493; doi:10.3390/gels9040265)
Supplement: Supplementary file 1 [file gels-09-00265-s001.zip › gels-2281061-supplementary.pdf]

Table S1

Primer sequence

M-α-Sma-F:	TCAGCAAACAGGAATACGACGA
M-α-Sma-R:	TTGGAAAGGAACTGGAGGCG
M-Col1a1-F:	TGTGTTCCCTACTCAGCCGT
M-Col1a1-R:	GTGCTTCTTTTCCTTGGGGTTC
M-Vegfa-F:	CTGGAAGAATCGGGAGCCTG
M-Vegfa-R:	AGGTGGGGTAAGGAGAGGAC
Mouse GAPDH-F	TGTGTCCGTCGTGGATCTGA
Mouse GAPDH-R	TTGCTGTTGAAGTCGCAGGAG
